# DNA Cleavage and Condensation Activities of Mono- and Binuclear Hybrid Complexes and Regulation by Graphene Oxide

**DOI:** 10.3390/molecules21070920

**Published:** 2016-07-15

**Authors:** Shuo Li, Mingxing Dai, Chunping Zhang, Bingying Jiang, Junqiang Xu, Dewen Zhou, Zhongwei Gu

**Affiliations:** 1 National Engineering Research Center for Biomaterials, Sichuan University, Chengdu 610064, China; lishuo@cqut.edu.cn; 2 School of Chemical Engineering, Chongqing University of Technology, Chongqing 400054, China; dai_mingxing@yeah.net (M.D.); jiangby@cqut.edu.cn (B.J.); xujunqiang@cqut.edu.cn (J.X.); dewen@cqut.edu.cn (D.Z.); 3 Jiangsu Key Laboratory of Target Drug and Clinical Application, Xuzhou Medical College, Xuzhou 221004, China

**Keywords:** cyclen, *N*,*N*′-bis(2-benzimidazolylmethyl)amine, DNA condenstion, DNA release, enzyme mimic, graphene oxide

## Abstract

Hybrid complexes with *N*,*N*′-bis(2-benzimidazolylmethyl)amine and cyclen moieties are novel enzyme mimics and controlled DNA release materials, which could interact with DNA through three models under different conditions. In this paper, the interactions between plasmid DNA and seven different complexes were investigated, and the methods to change the interaction patterns by graphene oxide (GO) or concentrations were also investigated. The cleavage of pUC19 DNA promoted by target complexes were via hydrolytic or oxidative mechanisms at low concentrations ranging from 3.13 × 10^−7^ to 6.25 × 10^−5^ mol/L. Dinuclear complexes **2a** and **2b** can promote the cleavage of plasmid pUC19 DNA to a linear form at pH values below 7.0. Furthermore, binuclear hybrid complexes could condense DNA as nanoparticles above 3.13 × 10^−5^ mol/L and partly release DNA by graphene oxide with π-π stacking. Meanwhile, the results also reflected that graphene oxide could prevent DNA from breaking down. Cell viability assays showed dinuclear complexes were safe to normal human hepatic cells at relative high concentrations. The present work might help to develop novel strategies for the design and synthesis of DNA controllable releasing agents, which may be applied to gene delivery and also to exploit the new application for GO.

## 1. Introduction

Artificial nucleases have attracted continuous and extensive interest due to their potential applications in the fields of molecular biology, biotechnology and drug development [1,2]. The design and synthesis of small-molecule catalysts as artificial nucleases for highly effective hydrolytic cleavage of the P–O bond in DNA are becoming a crucial tool in biotechnology. Metal complexes are the main part of artificial nucleases. Acting as Lewis acids, they stabilize the developing negative charge of the transition state and assist the departure of the leaving group [3]. Although the cations are the catalytic centers, the ligands are the key factors that influence the properties of artificial nucleases, and sometimes ligands participate in the catalytic process directly [4]. In general, nitrogen-containing compounds and their metal complexes display a wide range of biological activities, especially benzimidazole-substituted derivatives. For example, the benzimidazole moiety is the key constituent of vitamin B_12_ and is found in antibacterial, antifungal, antiviral [5,6,7] and antitumor agents as inhibitors of cyclin-dependent kinase and is useful for inhibiting cell proliferation [8,9]. *N*,*N*′-Bis(2-benzimidazolylmethyl)amine (IDB) derivatives, a kind of benzimidazole complex reported by our group recently, showed good antimicrobial properties and selective recognition toward cytosine [10].

At the same time, DNA protection and condensation are also fundamental life processes, and there are many studies in this field. Besides cationic polymers, cationic metal complexes are under evaluation as a kind of promising non-viral nucleic acid carrier. The early studies indicated that hexamine cobalt(III) cation (Co(NH_3_)_6_^3^^+^), a complex that was nonreactive to DNA, effectively induced DNA condensation [11]. Many other metal complexes which were used alone [12], as part of multicomponent systems [13] or conjugated with polymers [14] could deliver gene availability. Up to now, the design and synthesis of new cationic molecules for the purpose of controlling condensate size and morphology is still an important and attractive objective. Some novel complexes were developed, including AMD3100 polymer coordinated with Cu(II), [15] bis(zinc(II)-dipicolylamine)-functionalized material [16], acetylated 1,4,7,10-tetraazacyclotetradecane (cyclen) complex, acetylated IDB metal complexes [17] and [Ca(IDB)_2_]^2^^+^ derivatives [18]. Above all, cyclen, 1,4,8,11-tetraazacyclotetradecane, IDB, DTPB (1,1,4,7,7-penta(2′-benzimidazol-2-yl-methyl)-triazaheptane) and EGTB (*N*,*N*,*N*′,*N*′-tetrakis(2′-benzimidazol-2-yl-methyl)-1,4-bis(ethylamino)-bis-(ether)) are high performance ligands for small molecular DNA condensing agents. On the other hand, the release process of DNA nanoparticles also plays a crucial role in determining the effectiveness of the complex to deliver a therapeutic gene to the target cell nucleus. Cu^2+^-EGTB or its derivatives release genes through unknown mechanisms. Zhao and co-workers reported that azaheterocyclic-based metal complexes could release DNA controlled by temperature [17], and not through pH changing, “proton sponge” effect or compound degradation. Although acylation of cyclen reduced the cleavage properties of cyclen, the cleavage activity of the ring-Cu^2+^ complex would still result in a range of smaller DNA fragments at concentrations above 0.8 mM, and the condensation temperature was relatively high and the configuration of DNA changed by release [19].

To our best knowledge, there are few papers reporting hybrid complexes with cyclen and IDB and their bioorganic DNA interaction properties, so this attracted our interest. In the work presented here, we designed and investigated a serial of hybrid complexes composed of cyclen-ethyl and IDB-ethyl, and the results showed that there were synergetic effects and totally prior to their assemblies. However, few experimental studies have addressed how to modulate DNA condensation-release by convenient external additives besides matrix metalloproteinase [20] or GSH [21]. Graphene oxide (GO) was often used as a DNA cleavage promoter [22] or Cu^2+^/GO nuclease in some cases [23], and some kinds of cationic-modified graphene were used as gene delivery which release DNA by “proton sponge” effect [24]. At the same time, it is important to point out that the sp^2^-hybridized carbon backbone in GO maintains a high degree of planarity, which is much larger than that of most planar organic DNA intercalators or the aromatic ligands of inorganic DNA intercalators [25], so GO might offer a new kind controlled release method by π-π stacking, but there are few papers that report the direct use of GO was as regulation agent for gene release. Notably, this work was the first time using the classic planar aromatic structures of GO as a switch for DNA complexes.

Meanwhile, when these binuclear hybrid complexes were used as artificial nucleases in low concentration they showed interesting properties, but they were also seldom reported. To our surprise, the cleavage mechanisms of those complexes were different for their varied constructs, and some target materials cleaved DNA by an oxidation model and the others cleaved DNA by a hydrolysis model. Although several addition agents were also used to reduce the intensity of interactions between DNA and complexes, for example cyclodextrin was applied to wrap up naphthalene to avoid DNA breakdown [26] and GO is a newcomer to protect DNA when the concentration of target material is suitable for cleavage. From these results, it could be concluded that this kind novel hybrid compound could condense DNA in aqueous solution and that divalent metal ions enhance the efficiency of condensation. Importantly, GO may be used to regulate DNA condensation and release. The data is presented in this paper.

## 2. Results and Discussion

### 2.1. Concentration Effect of the Complex for DNA Cleavage and Condensation

**Scheme 1:**
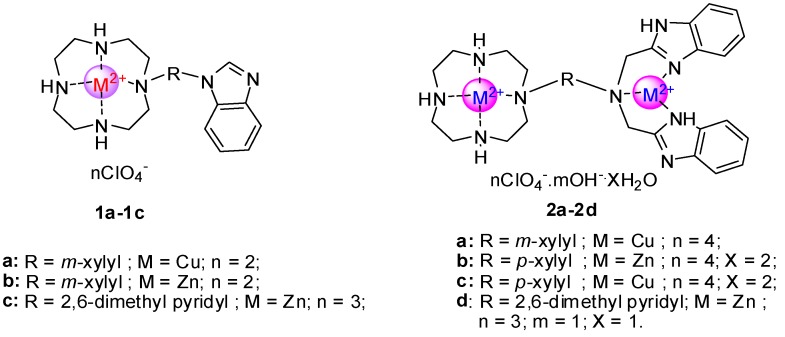
Structures of target benzimidazole complexes.

**Figure 1:**
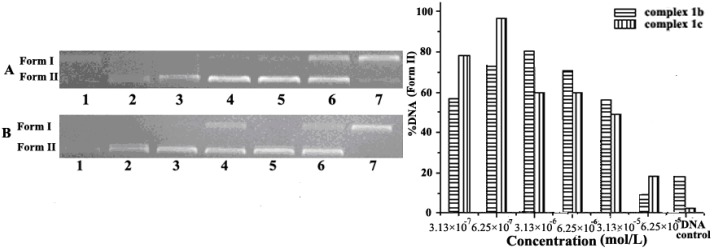
Histograms and electropherograms (the right side figure) representing cleavage of pUC19 plasmid DNA (0.008 µg/µL) by different concentrations of **1b** (pH = 7.4) and **1c** (pH = 7.4) in buffer (5 mM Tris-HCl/10 mM NaCl) at 37 °C for 6 h. (**A**) Complex **1b**; Lanes 1–6: 6.25 × 10^−^^5^, 3.13 × 10^−^^5^, 6.25 × 10^−^^6^, 3.13 × 10^−^^6^, 6.25 × 10^−^^7^, 3.13 × 10^−^^7^ mol/L, Lane 7 = DNA control, respectively; (**B**) Complex **1c**; Lanes 1–6: 6.25 × 10^−^^5^, 3.13 × 10^−^^5^, 6.25 × 10^−^^6^, 3.13 × 10^−^^6^, 6.25 × 10^−^^7^, 3.13 × 10^−^^7^ mol/L, Lane 7 = DNA control, respectively.

**Figure 2:**
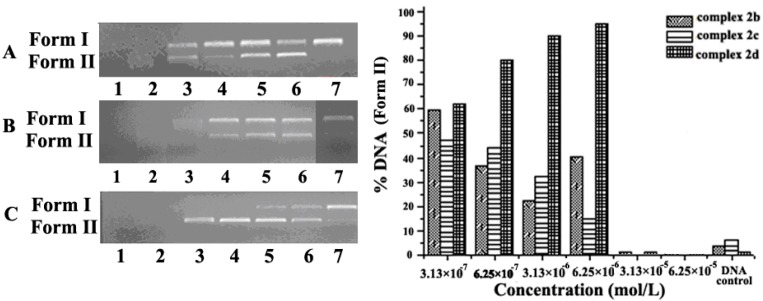
Histograms and electropherograms (the right side figure) representing cleavage of pUC19 plasmid DNA (0.008 µg/µL) by different concentrations of **2b** (pH = 7.4), **2c** (pH = 7.4) and **2d** (pH = 7.4) in buffer (5 mM Tris-HCl/10 mM NaCl) at 37°C for 6 h. (**A**) Complex **2b**; Lanes 1–6: 6.25 × 10^−^^5^, 3.13 × 10^−^^5^, 6.25 × 10^−^^6^, 3.13 × 10^−^^6^, 6.25 × 10^−^^7^, 3.13 × 10^−^^7^ mol/L, Lane 7 = DNA control, respectively; (**B**) Complex **2c;** Lanes 1–6: 6.25 × 10^−^^5^, 3.13 × 10^−^^5^, 6.25 × 10^−^^6^, 3.13 × 10^−^^6^, 6.25 × 10^−^^7^, 3.13 × 10^−^^7^ mol/L, Lane 7 = DNA control, respectively; (**C**) Complex **2d**; Lanes 1–6: 6.25 × 10^−^^5^, 3.13 × 10^−^^5^, 6.25 × 10^−^^6^, 3.13 × 10^−^^6^, 6.25 × 10^−^^7^, 3.13 × 10^−^^7^ mol/L, Lane 7 = DNA control, respectively.

DNA cleavage is controlled by relaxation of supercoiled form DNA (Form I). Plasmid pUC19 DNA, a widely used DNA cleavage substrate, is a circular double stranded DNA (Form I). The cleavage agent can convert Form I to nicked form (Form II) or linear form DNA (Form III). In gel electrophoresis, the migration rate of the three DNA forms is usually in the order: Form I > Form III > Form II. In order to assess the DNA cleavage activities of these complexes, the cleavage of plasmid pUC19 DNA assays were investigated by agarose gel electrophoresis. All these target compounds (structures shown in Scheme 1) were tested. Figure 1 and Figure 2, Figures S1 and S2 in the Supplementary Materials showed that the plasmid pUC19 DNA cleavage was promoted by mononuclear complexes at different concentrations from 3.13 × 10^−^^7^ to 6.25 × 10^−^^5^ mol/L, and pUC19 DNA degraded from Form I to Form II. Meanwhile, it must be pointed out that in Figure 1B form I would be convert to form II inconsistently by increasing the complex **1c**. We supposed that compounds with benzene rings would interact with each other strongly, so that the interactions between compounds would interfere with their cutting actions, but interferential effects were inconsistent upon increasing the complexes. To our surprise, increasing the complex concentration did not always result in more conversion of plasmid DNA from Form I to Form II. Furthermore, when the concentration approached 3.13 × 10^−^^5^ mol/L, some part of the DNA was condensed by the dinuclear complex, and its Form I and Form II bands became indefinite (shown in Figure 1 and Figure 2). Moreover, when the concentration became 6.25 × 10^−^^5^ mol/L, these bands nearly disappeared with both mononuclear complexes **1b**, **1c** and dinuclear complexes **2a**, **2b**, **2c** and **2d**. Taken together, these results indicate that the condensation interactions between DNA and dinuclear complexes are stronger than those of mononuclear complexes, and complex **2c** showed the strongest compression ability. The details are discussed below.

### 2.2. pH Effect on DNA Cleavage

**Figure 3:**
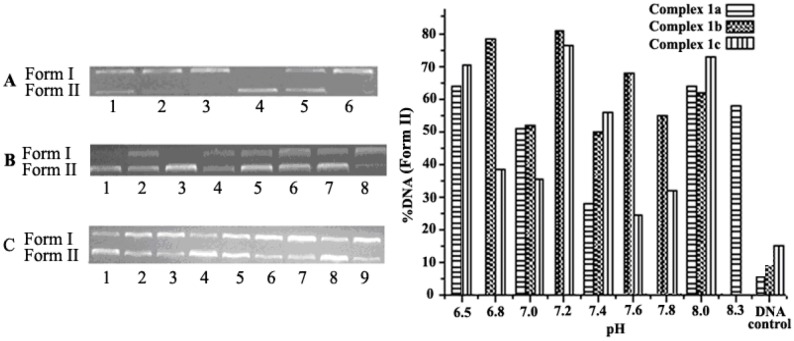
Histograms and electropherograms (the right side figure) representing cleavage of pUC19 plasmid DNA (0.008 µg/µL) in different pH buffer of **1a** (6.25 × 10^−7^ mol/L), **1b** (3.13 × 10^−6^ mol/L) and **1c** (6.25 × 10^−7^ mol/L) (5 mM Tris-HCl/10 mM NaCl) at 37 °C for 6 h. (**A**) Complex **1a**; Lanes 1−5: pH = 6.5, 7.0, 7.4, 8.0, 8.3, Lane 6 = DNA control, respectively; (**B**) Complex **1b;** Lanes 1–7: pH = 6.8, 7.0, 7.2, 7.4, 7.6, 7.8, 8.0; Lane 8 = DNA control, respectively; (**C**) Complex **1c;** Lanes 1–8: pH = 6.5, 6.8, 7.0, 7.2, 7.4, 7.6, 7.8, 8.0; Lane 9 = DNA control, respectively.

**Figure 4:**
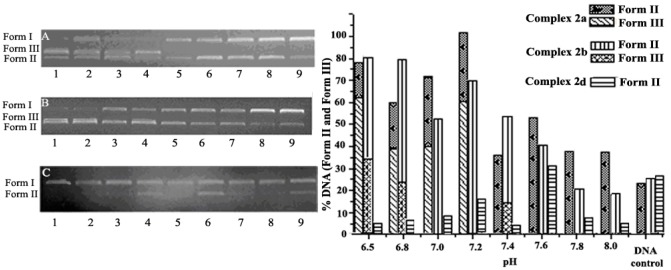
Histograms and electropherograms (the right side figure) representing cleavage of pUC19 plasmid DNA (0.008 µg/µL) in different pH buffer of **2a** (6.25 × 10^−7^ mol/L), **2b** (3.13 × 10^−7^ mol/L) and **2d** (6.25 × 10^−7^ mol/L) (5 mM Tris-HCl/10 mM NaCl) at 37 °C for 6 h. (**A**) Complex **2a;** Lanes 1–8: pH = 6.5, 6.8, 7.0, 7.2, 7.4, 7.6, 7.8, 8.0; Lane 9 = DNA control, respectively. (**B**) Complex **2b** and Lanes 1–8: pH = 6.5, 6.8, 7.0, 7.2, 7.4, 7.6, 7.8, 8.0 Lane 9 = DNA control, respectively; (**C**) complex **2d;** Lanes 1–8: pH = 6.5, 6.8, 7.0, 7.2, 7.4, 7.6, 7.8, 8.0; Lane 9 = DNA control, respectively.

pH-dependence profiles for DNA cleavage are shown in Figure 3 and Figure 4. From these figures, it can be seen that the pH-rate curve presents an “M-shape” (Figure 3) or ′′bell-shape′′ (Figure 4) profile with the acidity change of the solution of mononuclear and dinuclear complexes from pH = 6.5–8.3, and the optimal pH value for DNA cleavage is around 7.0 to 7.2 for both mononuclear and dinuclear complexes. This indicates that the cleavage efficiency of supercoiled DNA by complex is correlated to the pH value of the reaction system. In addition, the slightly alkaline conditions match humans’ normal physiological conditions, so major DNA cleavage assays in this work were performed in slightly alkaline solution (pH = 7.4). To our surprise, when the pH was lower than 7.0, dinuclear complexes showed stronger cleavage properties for Form III band formation, and mononuclear complexes had the same cleavage properties at pH below 7.0 as their cleavage properties at physiological conditions. It was proposed that for the benzimidazole group which contains two nitrogen atoms, one with pKa of 5.6 and another with a pKa of about 11, so the nitrogen atom with pKa = 11 could capture H^+^ and enhance the interaction with DNA with negative charge. It must be pointed out that the pKa of pyridine is 6.2, and the nitrogen atom in the pyridine could not be protonated at pH values from pH = 6.5 to 8.0. Furthermore, the linkage between cyclen and IDB greatly influenced the pH sensitivity of dinuclear complexes. For example, compounds **2a**, **2b** and **2d** were linked with a *m*-xylyl, *p*-xylyl and pyridyl group, respectively, and the pH-rate curves of compounds **2a**, **2b** or **2d** displayed different “M-shape” profiles (shown in Figure 4), the pyridyl group might participate in the coordination and reduce the Zn^2+^ activity to result in reduced cleavage ability regardless of the protonation of beneimidazole in IDB. As for compounds **2a** and **2b**, Form III is a linear form of DNA and showed that dinuclear complexes possess stronger cleavage properties at lower pH value.

### 2.3. Reaction Time Effect on DNA Cleavage

**Figure 5:**
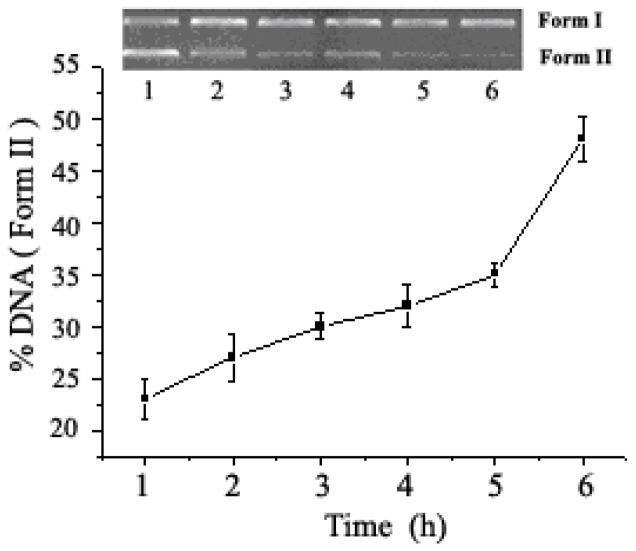
Time course of pUC19 DNA (0.008 µg/µL) cleavage promoted by **1b** (6.25 × 10^−7^ mol/L) in pH = 7.4 buffers (5 mM Tris-HCl/10 mM NaCl) at 37 °C. Lanes 1–6, reaction time 6, 5, 4, 3, 2, 1 h, respectively.

**Figure 6:**
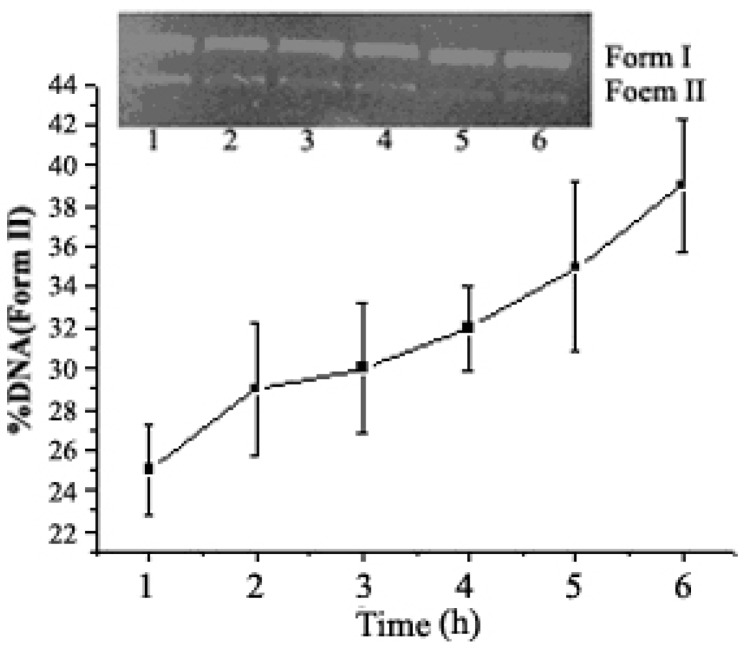
Time course of pUC19 DNA (0.008 µg/µL) cleavage promoted by **2b** (3.13 × 10^−7^ mol/L) in pH = 7.4 buffers (5 mM Tris-HCl/10 mM NaCl) at 37 °C. Lanes 1–6, reaction time 6, 5, 4, 3, 2, 1 h, respectively.

**Figure 7:**
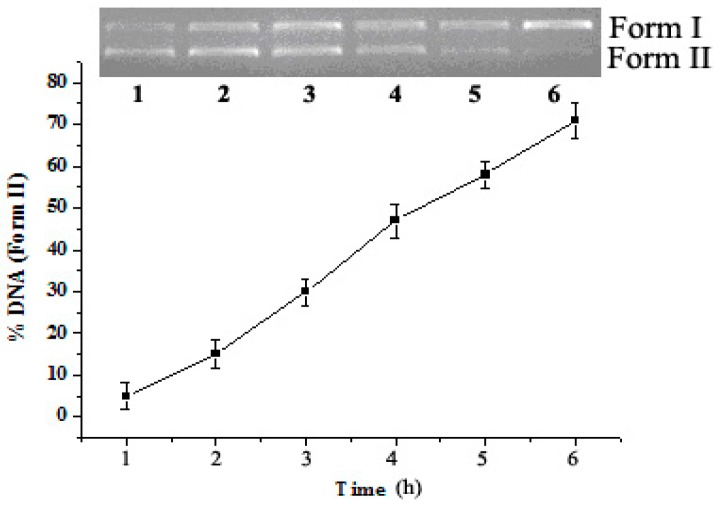
Time course of pUC19 DNA (0.008 µg/µL) cleavage promoted by **2c** (3.13 × 10^−7^ mol/L) in pH = 6.0 buffers (5 mM Tris-HCl/10 mM NaCl) at 37 °C. Lanes 1–6, 6, 5, 4, 3, 2, 1 h reaction time, respectively.

Agarose gel electrophoretogram of time courses of pUC19 DNA cleavage by complexes **1b** and **2b** and **2c** were selected for comparing ligands and cations, and results are shown in Figure 5, Figure 6 and Figure 7. These figures show that the longer the reaction time at 37 °C, the higher the conversion efficiency of plasmid DNA from Form I to Form II. The fluorescence intensity of Form II increases markedly with a corresponding decrease in the intensity of Form I. This result indicates that these complexes can promote the cleavage of plasmid pUC19 DNA from supercoiled Form І to linear Form II efficiently and the catalytic efficiency of DNA cleavage is correlated to the reaction time. From Figure 5 and Figure 6, it was found that both mononuclear complex and dinuclear complex would take more than 6 hours to cleave DNA, and the cleavage ability of dinuclear Zn^2+^ complex is weaker than that of mononuclear Zn^2+^ complex at the same Zn^2+^ concentration. Furthermore, from Figure 6 and Figure 7, it could be figured out that Cu^2+^ complex **2c** was more effective than Zn^2+^ complex **2b** at the same concentration and individual optimal cleavage pHs.

### 2.4. DNA Cleavage on the Presence of Typical Radical Scavengers

**Figure 8:**
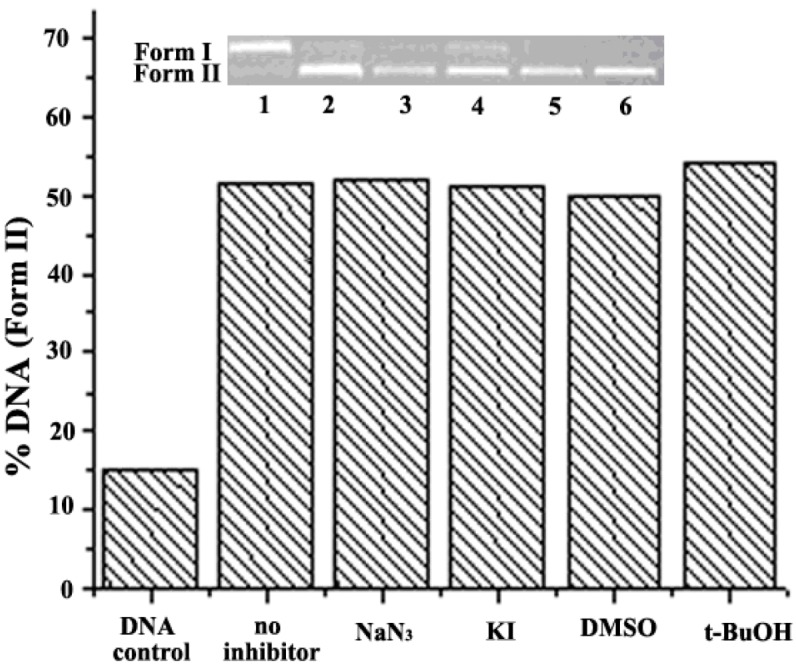
Histograms representing cleavage of pUC19 plasmid DNA (0.008 µg/µL) by compound **1a** with different typical radical scavengers (6.25 × 10^−7^ mol/L, pH = 8.0). Lane 1: DNA control, Lane 2: no inhibitor, Lane 3: NaN_3_, Lane 4: KI, Lane 5: DMSO, Lane 6: *t*-BuOH.

**Figure 9:**
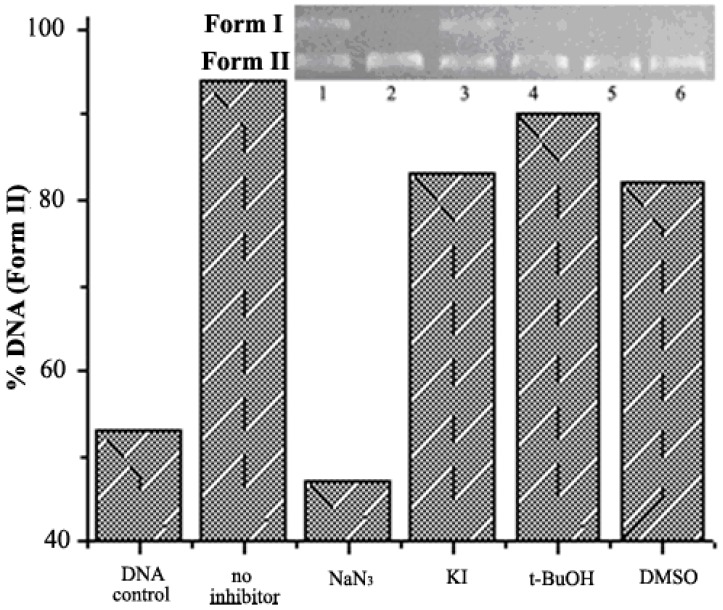
Histograms representing cleavage of pUC19 plasmid DNA (0.008 µg/µL) by compound **1b** typical radical scavengers (3.13 × 10^−4^ mol/L, pH = 7.4). Lane 1: DNA control, Lane 2: no inhibitor, Lane 3: NaN_3_, Lane 4: KI, Lane 5: *t*-BuOH, Lane 6: DMSO.

**Figure 10:**
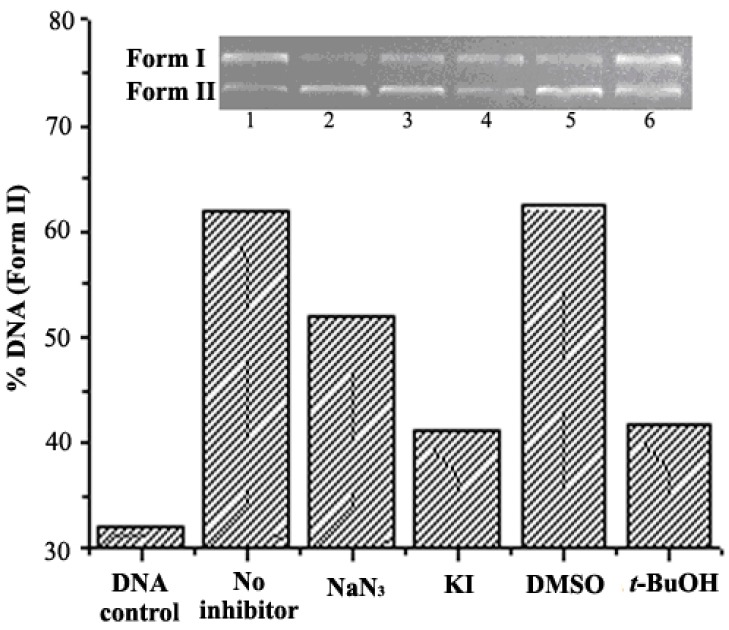
Histograms representing cleavage of pUC19 plasmid DNA (0.008 µg/µL) by compound **2a** typical radical scavengers (6.25 × 10^−7^ mol/L, pH = 7.4). Lane 1: DNA control, Lane 2: no inhibitor, Lane 3: NaN_3_, Lane 4: KI, Lane 5: DMSO, Lane 6: *t*-BuOH.

As is known, DNA cleavage generally proceeds via two major pathways: one is the hydrolytic pathway involving the phosphate group [4]; the other is oxidative cleavage of the sugar and/or nucleobase moiety due to the oxidation of the ribose or base group of DNA by reactive oxygen species resulting in the oxidative DNA cleavage pathway [27]. To explore the DNA cleavage mechanism by these complexes, the cleavage of DNA by mononuclear and dinuclear complexes was carried out in the presence of typical radical scavengers for singlet oxygen (NaN_3_), for superoxide (KI), and for hydroxyl radical (DMSO and *t*-BuOH) (shown in Figure 8, Figure 9 and Figure 10 and Supplementary Information). As evidently depicted in Figure 8, Figure 9 and Figure 10, in the presence of any of these scavengers (NaN_3_, DMSO, *t*-BuOH, KI), there were significant inhibition effects on the DNA cleavage by nearly all of these complexes, except for **1a**, which rules out the involvement of reactive oxygen species. Figure 8 shows that the cleavage property of complex **1a** was barely affected by these scavengers, which mean free radicals were slightly involved in the cleavage process, and that was to say DNA cleavage by **1a** was via a hydrolytic pathway, but other mononuclear compounds use oxidative cleavage to destroy DNA.

Figure 9 shows that sample **2a** with added NaN_3_ did suppress the cleavage efficiency, which was reduced over 60% by superoxide (KI) and hydroxyl radical (*t*-BuOH). Similar situations accompanied the cleavage processes of **2b**, **2c** and **2d**, and NaN_3_ and KI were the most effective individual suppressors. These results reflected that these target materials destroyed DNA via different processes and ligands affected these mechanisms and mononuclear Cu^2+^ complex **1a** had more probability to mimic hydrolytic enzymes. Furthermore, Cu^2+^ complexes always cleave DNA by an oxidative pathway, but the ligand structure was the key factor for artificial nucleases and some examples showed Cu^2+^ complexes could hydrolyse DNA for special ligands, such as diaza-crown ether [28] and 1,3-bis(1,4,7-triaza-1-cyclononyl)propane [29]. However, compounds with IDB always cleave DNA by an oxidative pathway expect for Fe^3+^ complexes, and it was proposed that these two centers of positive charge synergistically interacted with DNA units to destroy DNA for relative faster cleavage speed at the same cationic concentration. Therefore, the cleavage mechanisms of this kind of benzimidazole cyclen complexes were influenced by cations and IDB group, especially by the benzimidazole ligand.

**Scheme 2:**
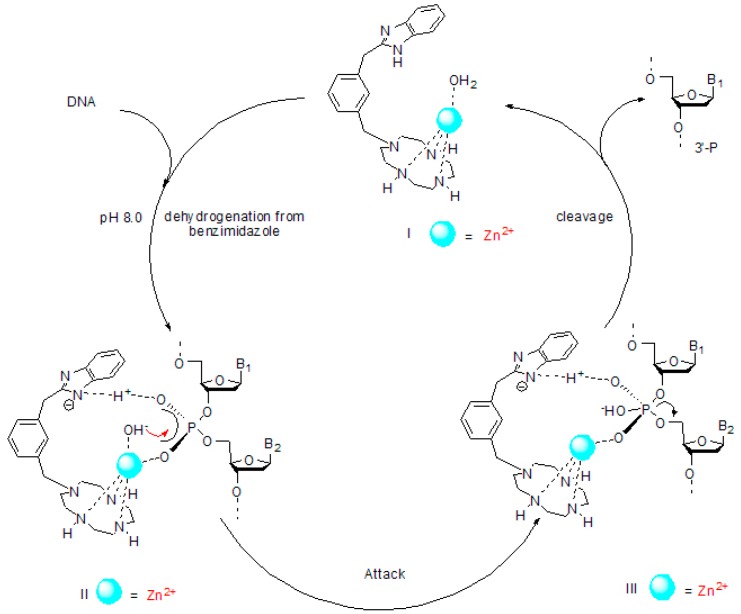
Possible mechanism of DNA cleavage catalyzed by the complex **1a**.

On the basis of the above DNA-cutting experimental results, a tentative mechanism of DNA cleavage catalyzed by the metal complexes **1a** as an example is proposed in Scheme 2. Like macrocyclic polyamine binuclear Zn^2+^ complexes [30] and Cu^2+^-IDB [31], H_2_O or oxygen atoms usually participate in coordination. With water in the unit of complex **1a** confirmed by elemental analysis [32], it was released that hydroxyl groups from crystallization water might be the attacking group at pH 8.0. This scheme indicated that the positive metal ion in the metal complex attracted negatively charged oxygen in the DNA phosphate group by electrostatic interaction, and then nucleophilic hydroxyl produced from water molecules associated with the metal ion attacked the phosphorus atom of DNA and then promoted the cleavage of P-O bonds to generate product.

### 2.5. DNA Condenstion and Regulation by GO

**Figure 11:**
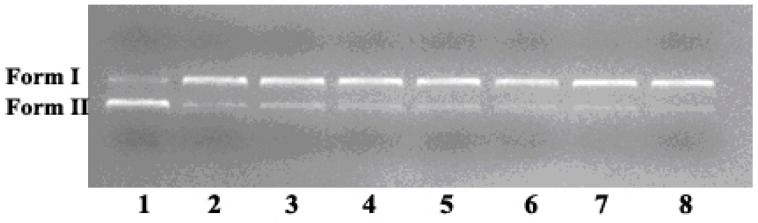
Electropherograms representing cleavage of pUC19 plasmid DNA (0.008 µg/µL) in buffer of different weight ratios of GO to complex **2c** (6.25 × 10^−7^ mol/L, 5 mM Tris-HCl/10 mM NaCl, pH = 6.0) at 37 °C for 8 h. Lanes 1–7: complex **2c** alone, 0.5, 1.0, 2.0, 3.0, 4.0, 5.0, respectively and Lane 8 is DNA control with GO (0.67 μg/μL), respectively.

Compound **2c** was selected to investigate the DNA condensation properties for its representative structure with the strongest compression ability according to Figure 2. At the same time, the particle sizes of **2c**/DNA complex with weight ratios 3.9 and 7.8 were 280 nm and 645 nm respectively. Meanwhile, the ξ potentials were 15 mV and 26 mV. Figure 11 reflects that GO alone could not condense or cleave DNA, and complex **2c** could effectively cleave DNA at 6.25 × 10^−7^ mol/L.

**Figure 12:**
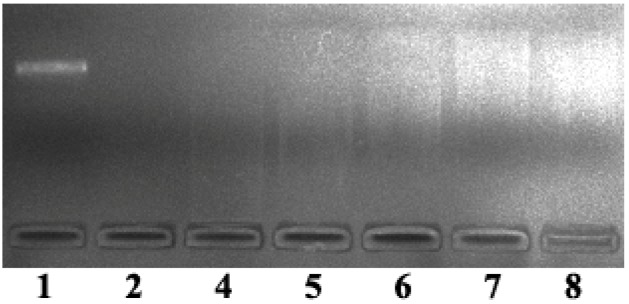
Electropherograms representing condensation and release of pUC19 plasmid DNA (0.008 µg/µL) in buffer of different weight ratios of GO to complex **2c** (5 mM Tris-HCl/10 mM NaCl, pH = 7.4) at 37 °C. Lane 1 is DNA control, Lanes 2–8: **2c** (6.25 × 10^−5^ mol/L), **2c** (3.13 × 10^−5^ mol/L), **2c** (6.25 × 10^−5^ mol/L)/GO (0.67 μg/μL), **2c** (6.25 × 10^−5^ mol/L)/GO (1.34 μg/μL), **2c** (3.13 × 10^−5^ mol/L)/GO (0.67 μg/μL), **2c** (3.13 × 10^−5^ mol/L)/GO (1.34 μg/μL), respectively.

However, when the weight ratio of **2c/**GO was above 0.5, the regulation function of GO was obvious and it inhibited the cleavage ability of **2c** to maintain most DNA in superhelical form. At the same time, Form I shown in Figure 12 disappeared when the concentration of **2c** was above 3.13 × 10^−5^ mol/L, but when GO was added after the DNA complex was formed, Form I was partly released. Furthermore, the amount of **2c** was important, and when the concentration was 6.25 × 10^−5^ mol/L, the release of DNA was weak, so the best control release concentrations of **2c** and GO were 3.13 × 10^−5^ mol/L and 1.34 μg/μL, respectively.

**Scheme 3:**
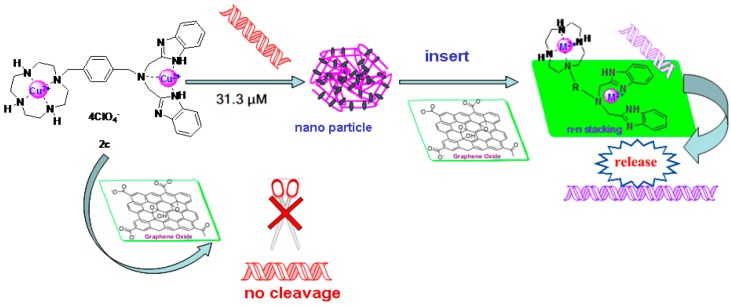
Possible mechanism of DNA release and cleavage blockage by complex **2c**.

As for GO regulation of the interaction between DNA and complex, the π-π stacking was the key factor, and complex adhered to GO by Cu^2+^-IDB which led to DNA release. Although the mononuclear compounds also have the benzimidazole group shown in Figure 1, they cannot condense DNA. Furthermore, cyclen complex conjugated with one or two big aromatic ring structures, such as anthracene [33] and acridine groups [34], always show DNA cleavage abilities. IDB had advantages over tridentate ligands like tris(benzimidazol-2-ylmethyl)amine (NTB) or ligands possessing four benzimidazole groups like *N*,*N*,*N*′,*N*′-tetrakis(benzimidazol-2-ylmethyl)-1,2-ethanediamine (EDTB), because of the internal angle between two benzimidazoles of Cu^2+^-IDB was nearly 180°, as verified by the single crystal structure [31] and the internal angles of benzimidazoles in NTB and EDTB were about 120° [18], so it was even greater in IDB than in NTB and EDTB. As we know, Cu^2+^-IDB and Cu^2+^-cyclen coordinated with one H_2_O each as confirmed by elemental analysis to maintain individual architecture stabilization, and the *p*-xylyl group as linker forced two nuclei away from each other, so there might be no interference between them. The benzyl group as linker also did not participate in coordination, and each cationic nucleus of the hybrid complex **2c** would keep its original configuration. Moreover, the benzyl linker might provide extra π-π stacking. It is also important to point out that the π-π stacking between two independent benzimidazoles has been proven [12] and the effect should be strong enough for GO to conjugate with dinuclear complexes. Figure 11 and Figure 12 also show that when complex **2c** and DNA were incubated in an EP tube together, no matter the DNA complex formed or approaching cleavage by electrostatic interaction, the GO could hinder the two interacting models. There was nearly no Form II or Form III DNA present and structures at the concentrations for cleavage were almost superhelical. Furthermore, it also can be found that the electrostatic interaction which is the key effect for commonly used gene delivery agents to condense DNA was relative weaker than the π-π stacking action. The possible mechanism of DNA release and cleavage hindrance by the complex **2c** is shown in Scheme 3.

To our surprise, the condensation concentrations of cyclen-ethyl and IDB-ethyl complexes at 2.0 × 10^−^^3^ mol/L were only about 11.1% and 12.3%, respectively (C_DNA_ = 3.1 × 10^−3^ µg/µL) [19]. However, most hybrid complexes at 3.13 × 10^−5^ mol/L (C_DNA_ = 8.0 × 10^−3^ µg/µL) could block nearly all superhelical DNA in gel as shown in Figure 12, and the condensation concentration was nearly 1/60 with regard to cyclen and IDB. From the results above, it can be concluded that there would be synergetic effects and the reinforcement between the two different cationic nuclei. This kind of unique synergetic structures of binuclear compounds might produce more stable condensation with DNA by stronger electrostatic interactions with two cationic nuclei. The DNA complex formed by acetylated cyclen at high temperature would disintegrate with decreasing temperature. [17] From the literature, it is also important to point out that the acetylated cyclen merged into the double-stranded of DNA and drew the outer sphere of DNA to form nanoparticles [17], so this kind of DNA complex formed with acetylated cyclen was negatively charged and zeta potentials were around −34 mV to −10 mV, and it was hard to adhere to the cells. In contrast, target materials could form complexes with positive charges which were blocked in the sample holes in the electrophoresis experiments could fulfill the needs of gene delivery.

### 2.6. Fluorescence Spectra of the Interaction between Complex and GO

**Figure 13:**
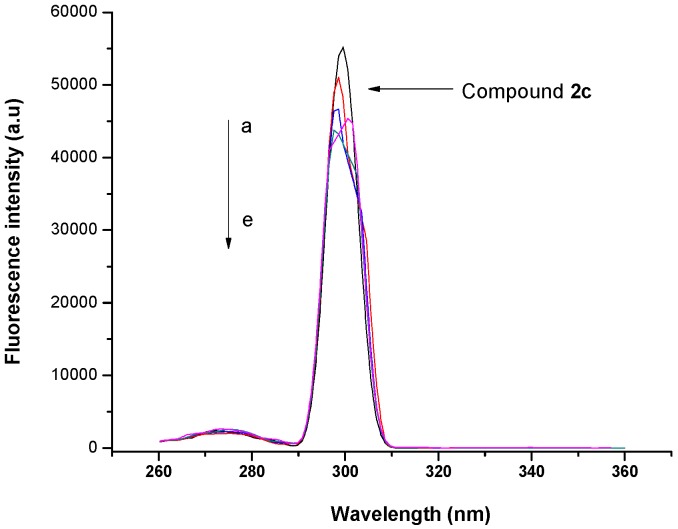
Emission spectra **a**–**e** the target material **2c** (1 × 10^−5^ mol/L in 5 mM Tris, 50 mM NaCl, pH = 7.4 buffer with increasing GO concentration (from 0 to 6.68 × 10^−5^ mg/mL).

Guo′s group recently demonstrated by using fluorescence spectroscopy the existence of single atomic layered graphene oxide (GO) sheets, due to their unique structural properties [35], so to further confirm the interactions between complex and GO, fluorescence spectra were used. From Figure 13, it could be seen that the peaks of emission spectra **a**–**e** were quenched by adding GO solutions. This confirmed that there was valid interaction between the GO and complex.

### 2.7. Cytotoxicity

**Figure 14:**
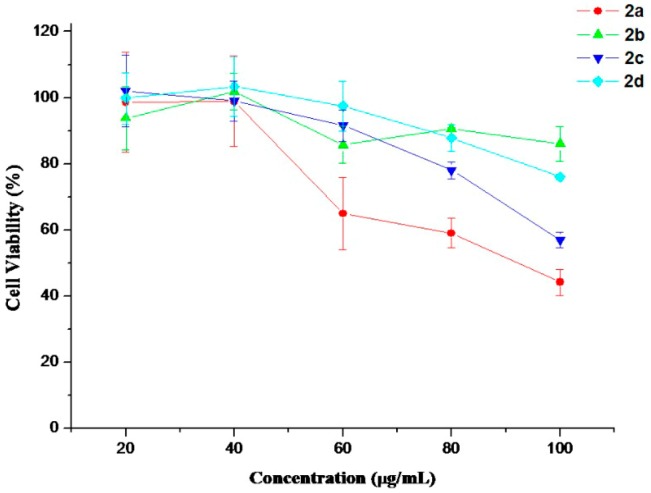
Relative cell viabilities of compounds **2a**–**d** in HL-7702 cells.

The cytotoxic activities of these complexes were tested by the CCK8 method, and evaluated in HL-7702 cells. As shown in Figure 14, none of them displayed serious cytotoxicity in this cell-line. The relative cell viability of **2b** or **2d** was more than 70% when the concentration was over 100 mg/mL. To our surprise, two Cu^2^^+^ complexes showed relative high cytotoxicity in the cell-line. Data obtained from in vitro and cell culture studies were largely supportive of copper′s capacity to initiate oxidative damage and interfere with important cellular events [36], but the results also reflected that all of these complexes **2a**–**d** showed relative low toxicity to normal human hepatic cells at high concentration, and dinuclear compounds were safe for further applications.

## 3. Experimental Section

### 3.1. Materials

Plasmid pUC19 DNA (TaKaRa Biotechnology, Dalian, China), 50 × TAE, 6× loading buffer, gold view dye, and agarose were purchased from Beijing Changsheng Biotechnology Co., Ltd. (Beijing, China). Cell Counting Kit-8 (CCK8) from Yeasen Company (Shanghai, China), trishydroxymethylamino-methane (Tris), HCl, NaCl, and NaOH were analytical grade products and used as supplied. All other chemicals purchased from Chongqing Chemical Co. (Chongqing, China), unless otherwise indicated, were of analytical grade. The water used for experiments was doubly distilled water. All chemicals were reagent grade and were used without further purification, unless otherwise noted. Graphene oxide solution was purchased from the Chinese Academy of Sciences Institute of Coal Chemistry (Taiyuan, China). 1-(3-((1,4,7,10-Tetraazacyclododecan-1-yl)methyl)benzyl)-1*H*-benzo[*d*]imidazole Cu^2+^ complex (**1a**), 1-(3-((1,4,7,10-tetraazacyclododecan-1-yl)methyl)benzyl)-1*H*-benzo[*d*]imidazole Zn^2+^ complex (**1b**), 1-((6-((1,4,7,10-tetraazacyclododecan-1-yl)methyl)pyridin-2-yl)methyl)-1*H*-benzo[*d*]imidazole Zn^2+^ complex (**1c**), *N*-(3-((1,4,7,10-tetraazacyclododecan-1-yl)methyl)benzyl)-*N*-((1*H*-benzo[*d*]imidazol-2-yl) methyl)-1-(1*H*-benzo[*d*]imidazol-2-yl)methanamine Cu(II) complex (**2a**), *N*-(4-((1,4,7,10-tetraazacyclododecan-1-yl)methyl)benzyl)-*N*-((1*H*-benzo[d]imidazol-2-yl)methyl)-1-(1*H*-benzo[*d*]imidazol-2-yl)methanamine Zn(II) complex (**2b**), *N*-(4-((1,4,7,10-tetraazacyclododecan-1-yl)methyl)benzyl)-*N*-((1*H*-benzo[*d*]imidazol-2-yl) methyl)-1-(1*H*-benzo[*d*]imidazol-2-yl)methanamine Cu(II) complex (**2c**) and 1-(6-((1,4,7,10-tetraaza-cyclododecan-1-yl)methyl)pyridin-2-yl)-*N,N*-bis((1*H*-benzo[*d*]imidazol-2-yl)methyl)methanamine Zn(II) complex (**2d**) were synthesized according to published procedures [10,32]. Because the solubilities of **1b**, **1c**, **2b** and **2d** were poor in doubly distilled water, so they were dissolved in DMSO to form 1 × 10^−3^ mol/L solutions, and then dissolved with doubly distilled water to get the test samples.

### 3.2. Instrumentation

The pH of the solution was determined by using a Sartorius PB-10 pH meter (Sartorius Scientific Instrument Co., Ltd., Beijing, China). The fluorescence spectra was recorded on a Lumina instrument (ThermoFisher, Waltham, MA, USA). The DNA cleavage was analyzed by gel electrophoresis on a DYY-12 electrophoresis meter (Beijing Liuyi Biotechnology Co., Ltd., Beijing, China) and a gel image analyzing system (Vilber Lourmat BIO-1D, Eberhardzell, Germany). Nano-ZS 3600 (Malvern Instruments, Westborough, MA, USA). Elix Advantage ultrapure water system (Merck Millipore, Darmstadt, Germany).

### 3.3. DNA Cleavage Experiments

The cleavage of pUC19 DNA was studied according to a reported procedure [28]. The cleavage in the presence of standard quenchers or promoters has also been investigated. In these experiments, DMSO (hydroxyl radical scavenger, final percentage 10%), 10 mM NaN_3_ (singlet oxygen scavenger), 10 mM KI (hydrogen peroxide scavenger) or was added to the solution containing SC DNA and complexes, respectively.

### 3.4. DNA Condensation, Cleavage and Regulation by GO Experiments

The condensation and regulation of pUC19 DNA was studied according to methods described before. Compound **2c** was selected as an example and the concentrations of **2c** were changed from 3.13 × 10^−^^5^ mol/L to 6.25 × 10^−^^7^ mol/L. As for DNA condensation and regulation experiments, DNA and compound **2c** were mixed and incubated for 20 min in room temperature before GO was added.

### 3.5. Fluorescent Spectrum Analysis

The interaction experiments were carried out according to the literature [22] with a Lumina instrument (Thermo Fisher) equipped with 1.0 cm quartz cells, the widths of both the excitation and emission slit were set as 5.0 nm, and the emission wavelength was 300 nm, connected to a temperature controller.

### 3.6. Particle Size and ξ Potential Measurements

Particle size and ξ potential measurements of polyplexes were carried out using a Nano-ZS 3600 (Malvern Instruments) with a He-Ne Laser beam (633 nm, fixed scattering angle of 901) at 25 °C. Complex **2c**/DNA at weight ratio were 3.9 and 7.8 prepared by the same method with abovementioned agarose gel electrophoresis. After 30 min incubation in 100 mL ultrapure water, polyplex solutions were diluted to a final volume of 1 mL before measurements.

### 3.7. Cell Viability Assay

Toxicity of **11a**,**e**,**f** toward HL-7702 cells was determined using a Cell Counting Kit-8 based on a (4-(3-(2-methoxy-4-nitrophenyl)-2-(4-nitrophenyl)-2*H*-tetrazol-3-ium-5-yl)benzene-1,3-disulfonate) WST-8 reduction assay following literature procedures [37]. The HL-7702 cells (6000 cells per well) were seeded into 96-well plates. The cells were then incubated in a culture medium containing **2a**–**d** with a particular concentration for 24 h. After that, 10 mL of CCK8 was added to each well. After 4 h, the unreacted dye was removed by aspiration. The OD value were measured spectrophotometrically in an ELISA plate reader (model 550, Bio-Rad, Hercules, CA, USA) at a wavelength of 450 nm. The cell survival was expressed as follows: cell viability = (OD treated/OD control) × 100%. The results are shown in Figure 14.

## 4. Conclusions

The studied novel benzimidazole mononuclear and dinuclear complexes exhibited nuclease activities towards DNA at low concentrations ranging from 3.13 × 10^−6^ to 6.25 × 10^−7^ mol/L. Compound **1a** showed almost similar DNA cleavage efficiency in the presence and absence of typical radical scavengers indicating that DNA cleavage by a hydrolytic cleavage mechanism. Most complexes showed good cleavage properties under lower pH as well as under physiological condition, and dinuclear complexes **2a** and **2b** can promote the cleavage of plasmid pUC19 DNA from supercoiled to the nicked and linear forms at pH = 7.0. It could be concluded that this kind of hybrid compounds could be used as novel artificial nucleases. More importantly, the most important functions of dinuclear compounds were to condense DNA, and this is the first time this novel method for DNA controlled release by GO is reported. Dinuclear compounds were relative safe for potential use in vivo.

## Supplementary Materials

Figures about reaction time effects of DNA cleavage of some target materials, figures of some complexes about DNA cleavage on the presence of typical radical scavengers, and figures to depict concentration effect of the complex for DNA cleavage and condensation. This material is available free of charge via the Internet. Supplementary materials can be accessed at: http://www.mdpi.com/1420-3049/21/7/920/s1.
