# Supplementary material for: DNA Cleavage and Condensation Activities of Mono- and Binuclear Hybrid Complexes and Regulation by Graphene Oxide
Source: Molecules. 2016 Jul 15;21(7):920. doi: 10.3390/molecules21070920 (PMC6274443; doi:10.3390/molecules21070920)
Supplement: Supplementary file 1 [file molecules-21-00920-s001.pdf]

Supplementary Materials: DNA Cleavage and Condensation Activities of Mono- and Bi-Nuclear Hybrid Complexes and Regulation by Graphene Oxide

Shuo Li, Mingxing Dai, Chunping Zhang, Bingying Jiang, Junqiang Xu, Dewen Zhou and Zhongwei Gu

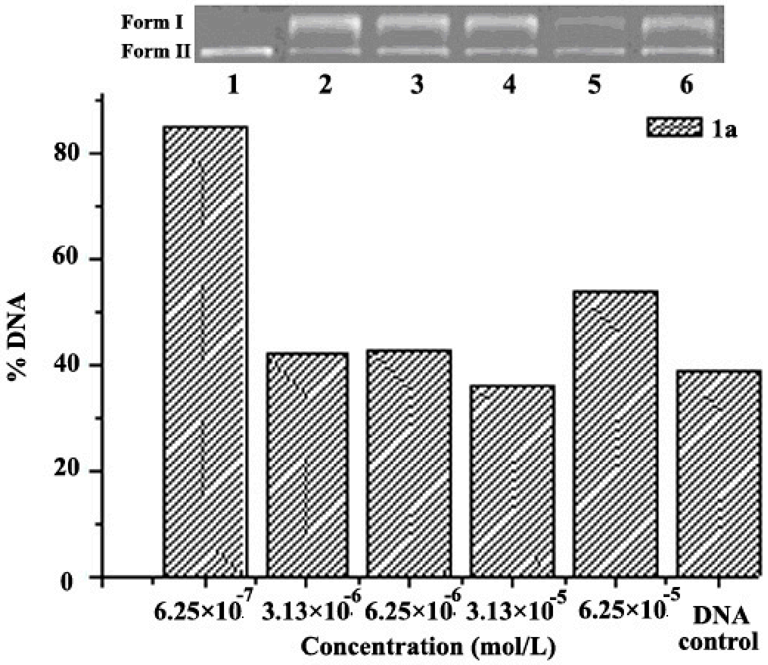

Figure S1. Histograms and electropherograms representing cleavage of pUC19 plasmid DNA (0.008 μg/μL) by different concentrations of **1a** (pH = 8.0) in buffer (5 mM Tris-HCl/10 mM NaCl) at 37 °C for 6 h. Complex **1a** and Lanes 1–5: complex **1a** and Lanes 1–5: 6.25×10^{-7}, 3.13×10^{-6}, 6.25×10^{-6}, 3.13×10^{-5}, 6.25×10^{-5} mol/L, and Lane 6 was DNA control respectively.

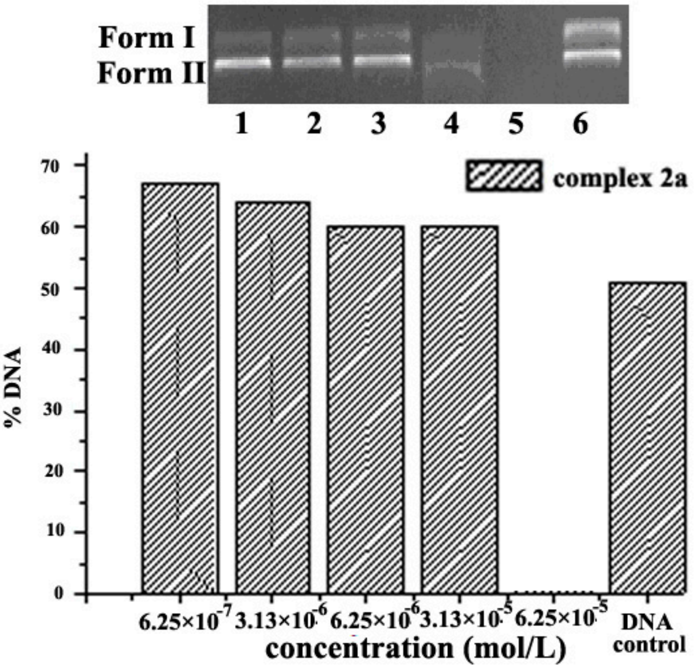

Figure S2. Histograms and electropherograms representing cleavage of pUC19 plasmid DNA (0.008 μg/μL) by different concentrations of **2a** (pH = 7.4) in buffer (5 mM Tris-HCl/10 mM NaCl) at 37 °C for 6 h. Complex **2a** and Lanes 1–5: 6.25×10^{-7}, 3.13×10^{-6}, 6.25×10^{-6}, 3.13×10^{-5}, 6.25×10^{-5} mol/L, and Lane 6 was DNA control respectively.

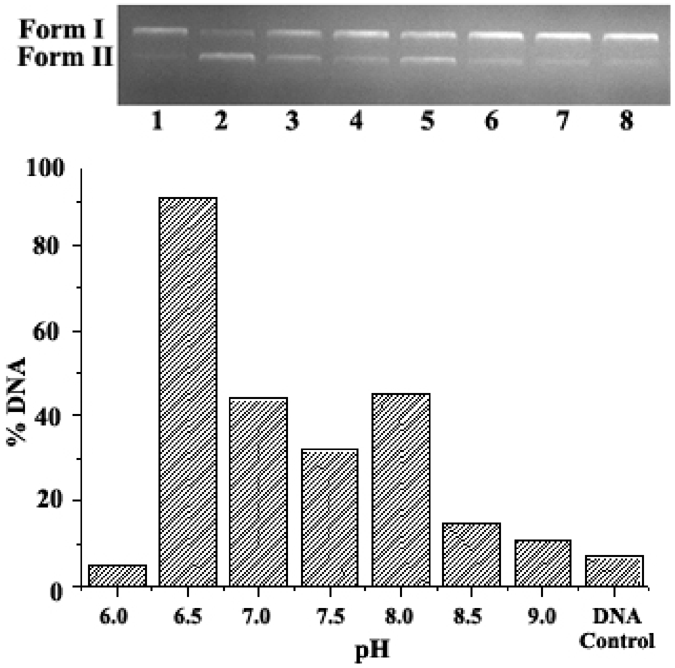

Figure S3. Histograms and electropherograms representing cleavage of pUC19 plasmid DNA (0.008 μg/μL) in different pH buffer of **2c** (6.25×10^{-7} mol/L) (5 mM Tris-HCl/10 mM NaCl) at 37 °C for 6 h. Lanes 2–8: pH = 6.0, 6.5, 7.0, 7.5, 8.0, 8.5, 9.0, and Lane 1 was DNA control respectively.

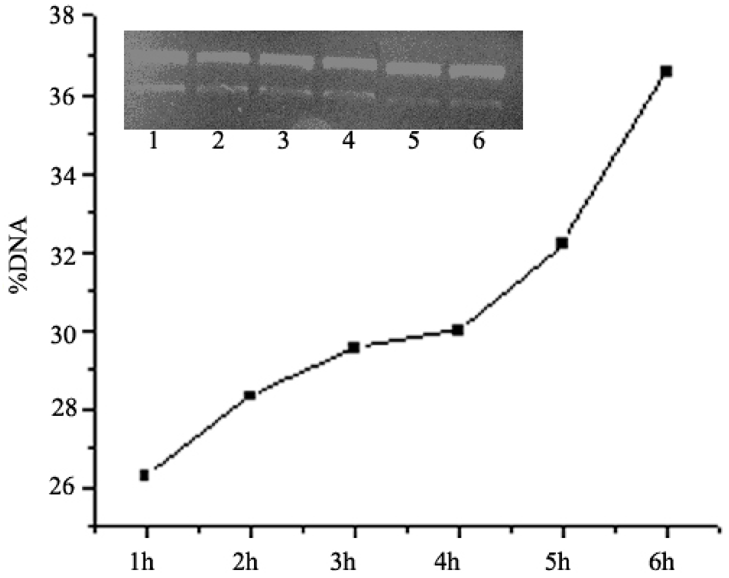

Figure S4. Time course of pUC19 DNA (0.008 μg/μL) cleavage promoted by **2b** (3.13×10^{-7} mol/L) in pH = 7.4 buffers (5 mM Tris-HCl/10 mM NaCl) at 37 °C. Lanes 1–6, reaction time 6, 5, 4, 3, 2, 1 h, respectively.

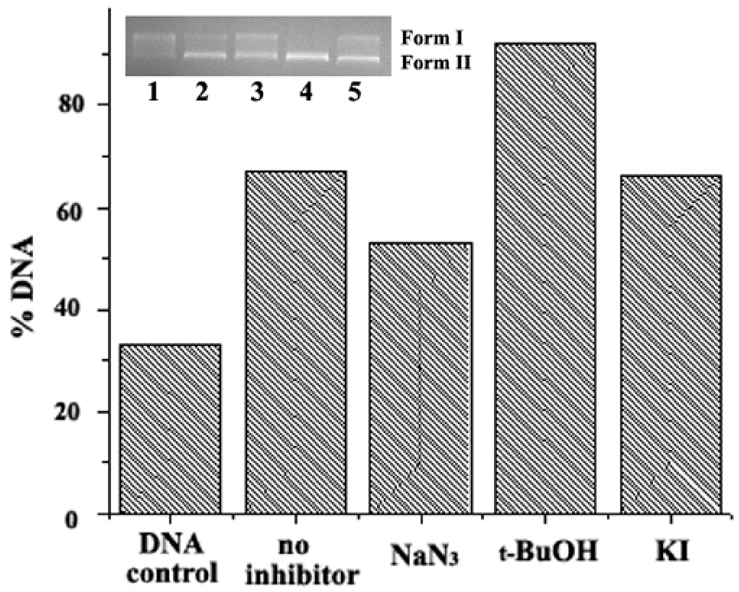

Figure S5. Histograms representing cleavage of pUC19 plasmid DNA (0.008 μg/μL) by compound **2b** typical radical scavengers (3.13×10^{-7} mol/L, pH = 7.4). Lane 1: DNA control, Lane 2: no inhibitor, Lane 3: NaN_3, Lane 4: *t*-BuOH, Lane 5: KI.

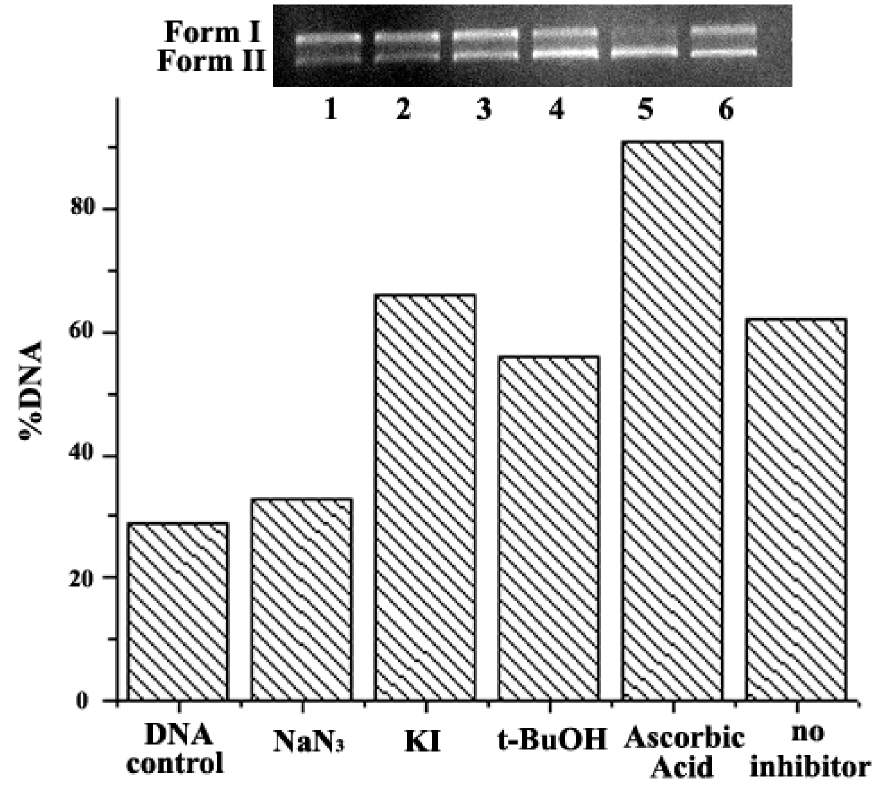

Figure S6. Histograms representing cleavage of pUC19 plasmid DNA (0.008 μg/μL) by compound **1c** typical radical scavengers (6.25×10^{-7} mol/L, pH = 7.4), Lane 1: DNA control, Lane 2: NaN_3, Lane 3: KI, Lane 4: *t*-BuOH, Lane 5: ascorbic, Lane 6: no inhibitor.
